# Glycosylator: a Python framework for the rapid modeling of glycans

**DOI:** 10.1186/s12859-019-3097-6

**Published:** 2019-10-22

**Authors:** Thomas Lemmin, Cinque Soto

**Affiliations:** 1 DS3Lab, System Group, Department of Computer Sciences, ETH Zurich, CH-8093 Zurich, Switzerland; 2 Institute of Medical Virology, University of Zurich (UZH), CH-8057 Zurich, Switzerland; 3 Vanderbilt Vaccine Center, Vanderbilt University Medical Center, Nashville, TN 37232 USA; 4 Department of Pediatrics, Vanderbilt University Medical Center, Nashville, TN 37232 USA

**Keywords:** *N*-linked glycosylation, Glycan modeling, Glycoprotein, Biomolecular modeling

## Abstract

**Background:**

Carbohydrates are a class of large and diverse biomolecules, ranging from a simple monosaccharide to large multi-branching glycan structures. The covalent linkage of a carbohydrate to the nitrogen atom of an asparagine, a process referred to as *N-*linked glycosylation, plays an important role in the physiology of many living organisms. Most software for glycan modeling on a personal desktop computer requires knowledge of molecular dynamics to interface with specialized programs such as CHARMM or AMBER. There are a number of popular web-based tools that are available for modeling glycans (e.g., GLYCAM-WEB (http://https://dev.glycam.org/gp/) or Glycosciences.db (http://www.glycosciences.de/)). However, these web-based tools are generally limited to a few canonical glycan conformations and do not allow the user to incorporate glycan modeling into their protein structure modeling workflow.

**Results:**

Here, we present Glycosylator, a Python framework for the identification, modeling and modification of glycans in protein structure that can be used directly in a Python script through its application programming interface (API) or through its graphical user interface (GUI). The GUI provides a straightforward two-dimensional (2D) rendering of a glycoprotein that allows for a quick visual inspection of the glycosylation state of all the sequons on a protein structure. Modeled glycans can be further refined by a genetic algorithm for removing clashes and sampling alternative conformations. Glycosylator can also identify specific three-dimensional (3D) glycans on a protein structure using a library of predefined templates.

**Conclusions:**

Glycosylator was used to generate models of glycosylated protein without steric clashes. Since the molecular topology is based on the CHARMM force field, new complex sugar moieties can be generated without modifying the internals of the code. Glycosylator provides more functionality for analyzing and modeling glycans than any other available software or webserver at present. Glycosylator will be a valuable tool for the glycoinformatics and biomolecular modeling communities.

## Background

Glycosylation is an important post-translational modification of proteins, where a carbohydrate is covalently attached by an enzyme to specific amino acids motifs known as sequons space [1–4]. Glycosylation has several principal structural and functional roles in biology, that include protein folding [5], tissue repair [6], and cell migration [7]. In eukaryotes, nearly 70% of the proteome is believed to be glycosylated [8]. More recently, glycosylation has been observed in bacteria where it has been associated with their virulence and the formation of biofilms [9]. For viruses, such as HIV and Influenza, glycosylation allows for evasion of the host’s immune system [10, 11]. Thus, determining the role of glycan structure in biology is essential in order to understand pathogenesis. The diverse and dynamic nature of glycan structures makes it difficult to resolve their structure experimentally through traditional approaches (e.g., x-ray crystallography, cryogenic electron microscopy (cryo-EM) or nuclear magnetic resonance (NMR)). Computational methods, such as molecular dynamics (MD) can help resolve glycan dynamics but this method is computationally intensive and cannot be used for the rapid modeling of glycan structure. Complementary techniques that are more rapid and available through a graphical user interface (GUI) should allow users to gain new insights into glycan-protein structure.

In silico modeling of glycoprotein is a tedious and time consuming process and tools, such as CarbBuilder [12], POLYS [13], doGlycans [14], SWEET-II [15], GLYCAM-Web [16], Glycan Reader [17, 18] and CHARMM-GUI glycan modeler [19] were developed to facilitate the modeling of glycans. CarbBuilder, POLYS and doGlycans are open source programs that allow building glycan structures from their primary sequence of monosaccharide units. SWEET-II is part of the website Glycosciences.DB [20] and can be used to build 3D structures of glycans. Furthermore, the website provides a number of tools for manipulating and analyzing glycans. GLYCAM-Web offers several options that simplify the building and set-up of molecular dynamics simulations of glycoproteins. It uses the GLYCAM force field [21] that is compatible with the AMBER force field. Finally, Glycan Reader recognizes most types of glycans and their chemical modifications found in the Protein Data Bank (PDB), which are all available in the CHARMM force field [22]. It also provides the option for editing their three-dimensional structure. Glycan Modeler generates complex glycans and glycoconjugates by searching templates from a fragment database. Glycan Reader and Modeler have both been integrated into CHARMM-GUI [23], a powerful website widely employed for setting up molecular dynamics simulation. In addition, CHARMM-GUI provides the functionality for modelling glycolipids and lipopolysaccharides (LPS) and to combine them with complex biological membrane simulations [24]. While many of these tools are available as webservers making them ideal for their ease of use and distribution, this limits their ability to be customized for the specific needs of some users; for example, for tasks that require batch modeling of several glycoforms for a given protein or adding non-canonical saccharides to a protein structure.

We describe here Glycosylator, a Python framework designed for the rapid modeling of glycoprotein. It can be used directly in a Python terminal or script to identify, manipulate, and build glycans. In addition, the GUI allows for the quick visualization and modification of glycosylated proteins (such as one downloaded directly from the PDB). The molecular description of glycans is based on the CHARMM force field [22]. New saccharides appearing in updated versions of the force field or defined by the user can easily be added. Modeled glycans can be further refined by removing clashes and sampling alternate conformations. Since Glycosylator is distributed as a Python package, users can easily adapt the code to meet their specific needs.

## Implementation

The Glycosylator framework is composed of 7 classes, several of which can be employed as standalone instances for other applications in molecular modeling (Additional file 1: Figure S1 in the Supporting Information (SI) section). At the core of Glycosylator is the Molecule class. A Molecule is defined as a single covalently linked set of atoms and is implemented around the ProDy [25] and NetworkX [26] packages. ProDy is widely used for studying biomolecules and offers several functions for storing and manipulating structures. The functions and classes provided are used in the Molecule class for saving and quickly accessing the structural data of a molecule. The topological properties of a molecule are represented here as a graph using the NetworkX package. A Molecule can be instantiated directly with a 3D structure (PDB) or using a MoleculeBuilder instance and the topology information provided for the CHARMM force field [22]. When loading a glycoprotein, Glycosylator will identify all *O-* and *N-*linked sequons and their glycans. The structure and topology of each of the glycans can then be modified. Clashes and alternative conformations for glycans can be optimized with the Sampler class. Finally, the graphical representation of glycans provided by the Drawer class makes use of Matplotlib [27], a Python package used for plotting. Taken together, Glycosylator provides more functionality for analyzing and modeling glycans than many popular software packages and webservers (Table 1). The main functions used for glycosylating a protein can be conveniently accessed through Glycosylator’s GUI (Additional file 1: Figure S2).

**Table 1:**
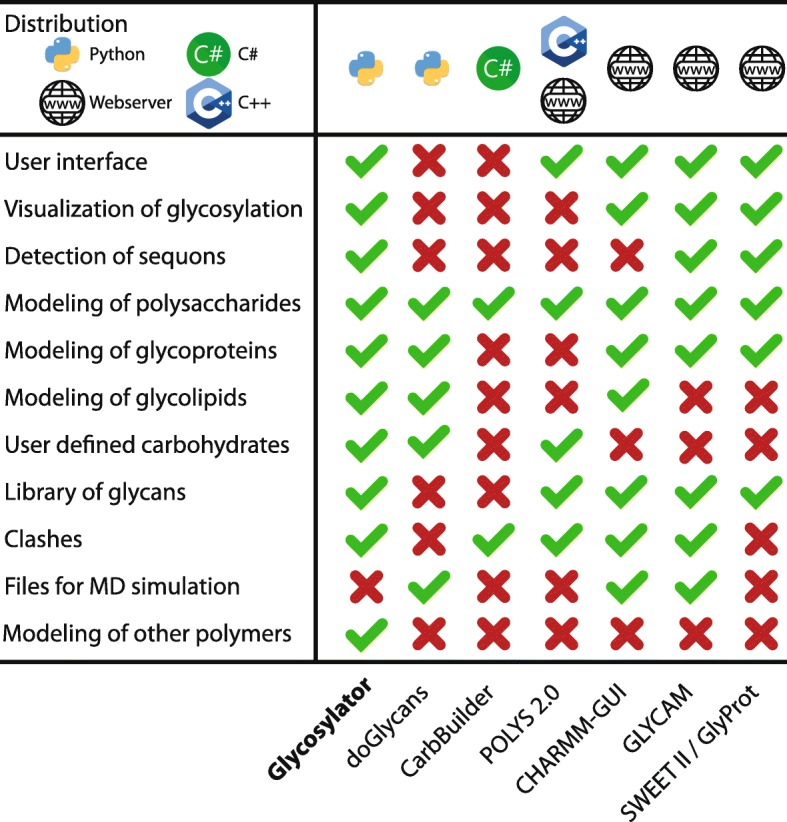
List of functionalities offered by the available software and webservers for modeling glycans. CHARMM-GUI includes Glycan Reader and Modelers, as well as the glycolipid and LPS modelers

Below, we briefly describe each class. Detailed examples for the usage of each class are provided in the Supporting Information (Additional file 1: Example S1) section and on the Github repository.

### CHARMM classes

CHARMM force field topology and parameter files are parsed using the CHARMMTopology and CHARMMParameters classes, respectively. The data is stored in a dictionary for a quick and easy access. The CHARMMTopology class creates and stores an additional dictionary for looking up patches. The patches are used to define the glycosidic bonds between saccharide units and are required for modification (e.g., deleting atoms).

### Molecule class

The Molecule class is used for storing the coordinates (Prody’s AtomGroup) and connectivity (NetworkX graph) of a molecule. The bonds, angles and dihedrals are assigned either by the user or automatically based on the distances between atoms. The molecule’s connectivity is saved as a directed graph. The user can provide the root atom to define the direction of the connectivity graph; by default, the first atom of the molecule is chosen. Ring structures are automatically detected identifying all rotatable torsional angles that are not part of a cycle. These torsional angles can be measured, set to a specific value or rotated by a given amount. An inter-residue graph is also built in order to quickly parse through a molecule composed of several residues.

### MoleculeBuilder class

The MoleculeBuilder class is employed for building and editing molecules. Information about the connectivity and atoms of a molecule are extracted from a CHARMMTopology instance. This class allows for the initialization of a Prody residue (AtomGroup). Applying a patch (CHARMM) will modify one or several residues. For glycans, patches are typically used to define the glycosidic linkage. MoleculeBuilder interfaces directly with the Prody AtomGroup and returns all the information required for creating a Molecule instance.

### Glycosylator class

Glycosylator class was designed to deal specifically with glycans/glycoprotein. It can import a PDB file and automatically extract all the *O-* and *N-*linked sequons and associated atoms. Each glycan is saved as a Molecule instance in a dictionary. The key of the dictionary is the residue number and chain of the sequon. Glycosylator uses an internal text representation for storing a topology tree for each glycan structure. These trees describe the connectivity and saccharide units that compose a polysaccharide. A library of these structures can be imported into a Glycosylator instance or saved as a simple text file or a SQL database. Glycosylator can then compare the extracted connectivity tree to the internal dataset of known glycans to identify them based on the glycosidic linkage and residue type. We do note that chemical post-modifications of glycans are not supported in the current version. Glycans can be extended, trimmed or modeled ab initio. This can be achieved by providing the identification of a known oligosaccharide (in the library) or with a topology tree describing the connectivity and glycan units of the desired oligosaccharide. The topology tree is a string representation of a glycan.

### Sampler class

Sampler class implements a genetic algorithm for removing clashes between Molecules and their environment (e.g., protein). The CHARMM force field energy function for the torsional angles will be used for biasing the random number generator and to sample more energetically favorable torsional angles [22]. The generation of the initial population can be skewed towards the common co-dependence of angles. The fast clash detection algorithm is based on K-d trees for intra- and inter-clashes of glycans. Standard grid mapping is used for the detection of clashes between glycans and their environment. To reduce the search space, the genetic algorithm iteratively optimizes subsets of glycans with the highest number of steric clashes.

### Drawer class

Drawer class is used for generating 2D symbolic representations of glycans according to the IUPAC standard. The inter-residue connectivity graph stored in a Molecule is used for drawing the connectivity of a glycan. The protein is represented as a ribbon, each sequon is highlighted and the linked glycans are shown as a tree topology. The graphical representation is produced with Matplotlib and can be further modified by the users (e.g., add text, rescale) and exported in various image formats.

## Results

### Benchmark on viral glycoproteins

We compared the performance of Glycosylator and doGlycans, another Python framework for modeling glycans using three representative viral envelope glycoproteins, each containing different numbers of glycosylation sites and overall glycan density. The glycans on the surface of these proteins create a shield that helps them to evade the host’s immune system [28]. For the benchmark, a mannose 9 was modeled at each sequon, mimicking the glycosylation state before exiting the endoplasmic reticulum [29]. The topology of the glycosylated structure was generated with the autopsf plug-in of VMD [30]. Each glycoprotein was then minimized with 5000 steps of conjugate gradient optimization in NAMD [31]. The resulting energy-minimized model was then submitted for a sanity check to pdb-care (http://www.glycosciences.de/tools/pdb-care/), a powerful tool that checks the connectivity and nomenclature in glycoproteins [32]. We observed that all glycoproteins modeled with Glycosylator had a lower potential energy and were devoid of any steric clashes and topological errors (Table 2). For structures with a low density of sequons, such as Influenza’s hemagglutinin, Glycosylator and doGlycans performed similarly. However, a simple minimization was insufficient for removing steric clashes from the HIV-1 Envelope trimer and Delta coronavirus spike protein structures using doGlycans. The density of sequons at the surface of these glycoproteins is high, requiring a more effective strategy for removing clashes, such as provided by Glycosylator’s Sampler Class. The steric clashes present in the structures produced with doGlycans lead topological errors, such as ring puckering after minimizations. In order to solve this issue, the torsional angles would have to be manually adjusted by the user.

**Table 2:** Benchmark comparing Glycosylator and doGlycans. The average minimum distance between sequons was computed between the closest pairs of asparagine Cα atoms. The number of issues accounts for errors in glycan connectivity and nomenclature due to steric clashes. The potential energy was calculated after 5000 steps of conjugate gradient energy minimization

Virus		Influenza A	HIV-1	Delta-coronavirus
PDB id		1 ha0	5fyl (gp120)	6bfu
Number of sequons		6	20	21
Average minimum distance between sequons	[Å]	21.60 ± 8.88	10.47 ± 4.74	14.71 ± 4.11
**Glycosylator**	Number of issues	0	**0**	**0**
	Potential energy [kcal/mol]	**− 9856.12**	**2524.05**	**− 5526.44**
DoGlycans	Number of issues	0	4	6
	Potential energy [kcal/mol]	− 9678.14	6055.20	− 1954.89

Better performance are highlighted in bold

### Identifying and batch modeling *N*-linked glycans onto the HIV-1 Env trimer

**Fig. 1:**
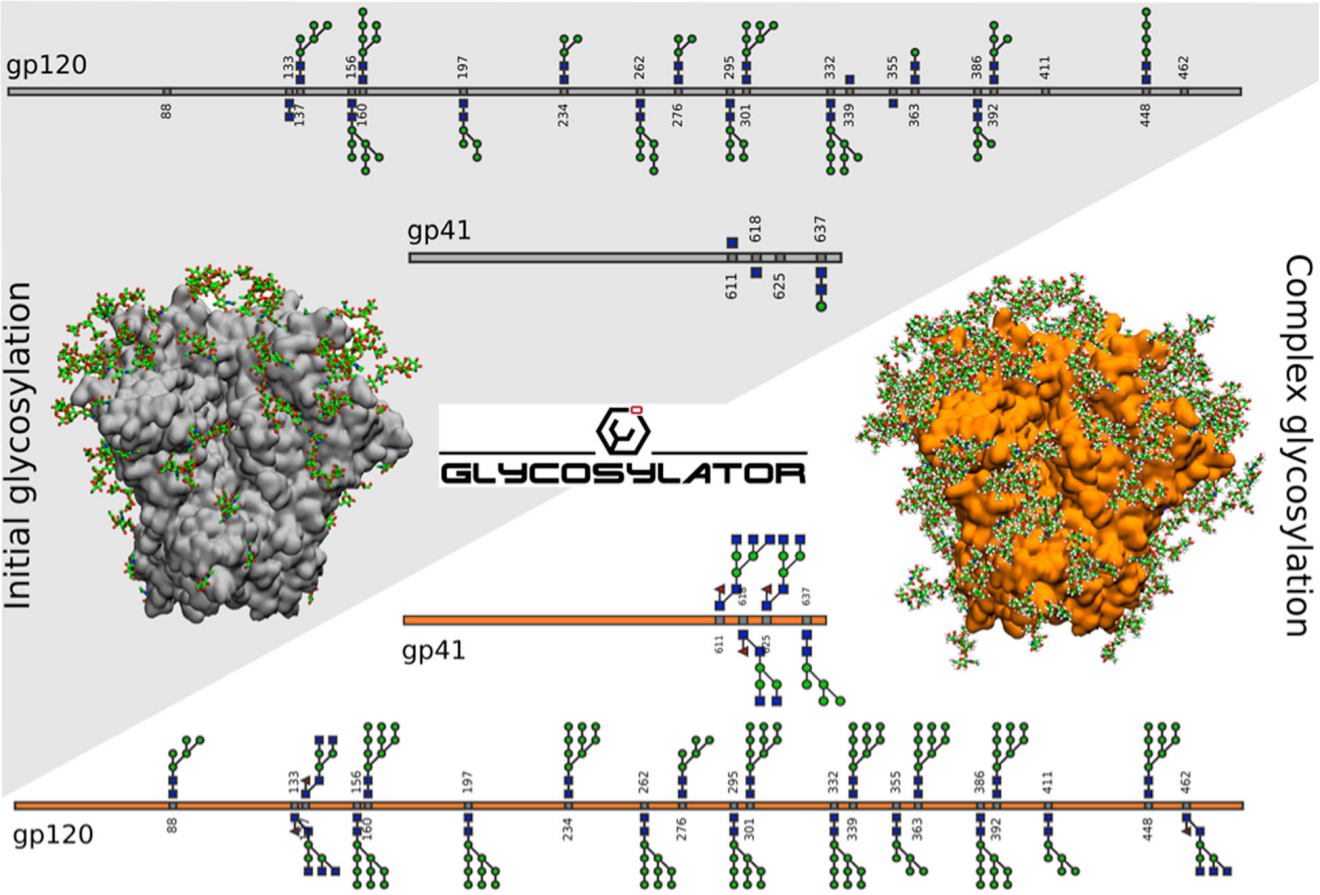
Identification, visualization and modeling of N-linked glycans onto the HIV-1 Env trimer. Protein surface representation of the high-mannose glycoform of the HIV-1 Env Trimer (PDB ID: 5fyl). Crystallographically-determined glycans are shown in Licorice representation. Each subunit (gp120 and gp41) is represented as a ribbon with sequons indicated with gray squares and the N-linked glycans shown above or below the sequon (upper left triangular panel). Glycosylator was used to produce a complex glycoform variant of the HIV-1 Env trimer by modeling glycans ab initio or extending existing glycans (lower right triangular panel)

As an additional test case, we modeled the glycan shield of the HIV-1 Env trimer using Glycosylator. The HIV-1 Env trimer consists of 80–100 sequons making it one of the most highly glycosylated proteins currently known. We chose the BG505-SOSIP structure with PDB: ID 5fyl, [33]) as the starting structure. First, all crystallographically-determined glycans were identified and hydrogenated (Fig. 1, upper left triangle). The ribbon representation allowed for a quick visual inspection of the identified *N-*linked sequons and linked glycans. A combination of mannose 5, mannose 9 and complex glycans was then modeled ab initio or by extending existing glycans to produce a more biologically relevant glycoform of the HIV-1 Env trimer (Fig. 1, lower right triangle). The Sampler function in Glycosylator was then used to remove all major clashes, such that the topology of the full glycoprotein could be generated directly with the autopsf plug-in of VMD [30]. The remaining clashes were quickly removed with 5000 steps of conjugate gradient energy minimization in NAMD [31]. The resulting model was then submitted to the pdb-care server [32] for a sanity check and we found no discrepancies in connectivity. The Python script used for this example is available in the GitHub repository. Two additional examples for building and identifying glycans can be found in the Supporting Information section (Additional file 1: Examples S1 and S2).

## Conclusion

Glycosylator is a versatile Python framework for manipulating glycans and glycoproteins that facilitates the structural study of glycans. It will significantly improve the ability of the glycobiology community to model glycan structure without requiring advanced expertise in protein modeling or molecular dynamics. Glycosylator has already been successfully used for several studies investigating the dynamics of glycans over long timescales (500 ns to 2 μs) [33–35]. Glycosylator is a valuable asset for glycoinformatics and biomolecular modeling communities. Furthermore, it should be noted that Glycosylator can also be used to model other polymers (D09_polymer in Github).

## Availability and requirements

**Project name:** Glycosylator.

**Project home page:** https://github.com/tlemmin/glycosylator

**Operating system(s):** Platform independent.

**Programming language:** Python.

**License:** MIT.

## Supplementary information

**Additional file 1: Figure S1.** Architecture of Glycosylator, a Python framework for the rapid modeling of glycans. Each class is represented by a hexagon. Full circles connecting classes indicate a class that contains an instance of the previous one as an attribute, e.g. instances of Molecule and MoleculeBuilder are attributes of Glycosylator. Several attributes from Glycosylator can be directly shared with Drawer and Sampler (white squares). Glycosylator can parse a PDB file of a glycoprotein and identify all the sequons (orange rhombus). The glycans (blue squares and green circles) will be extracted and saved as Molecule instances. Glycans at each sequon can then be built, modified or identified. **Figure S2.** Glycosylator Graphical User Interface. a) The main window is used to import a PDB file of a glycoprotein. Glycosylator will produce a symbolic representation (orange dashed line rectangle). A specific sequon can be selected in the right panel (purple dashed line rectangle). The glycan can be modified by clicking on the symbolic representation. b) The user can select a glycan from the common library or a library that they created. The selected glycan is highlighted with a red square. **Example S1.** Building a glycan. The structure of an N-Acetyl-D-Glucosamine will be imported as a Molecule instance. All missing atoms will be added according to the CHARMM force field. A second N-Acetyl-D-Glucosamine will then be linked through a 1-4 glycosidic bond. Finally, an Alpha-D-added according to the CHARMM force field. A second N-Acetyl-D-Glucosamine will then be linked through a 1-4 glycosidic bond. Finally, an Alpha-D-Mannosewill be built ab initio and saved to a PDB file. **Example S2.** Importing, identifying and editing of a glycan. The structure of a mannose 9 will be imported as a Molecule instance. A Glycosylator instance will then identify it against a database of known structures. Finally, the Molecule will be trimmed down to a mannose 6.

## Data Availability

Glycosylator is available from the following Github repository: https://github.com/tlemmin/glycosylator

## Abbreviations

API: Application Programming Interface
GUI: Graphical User Interface
NMR: Nuclear Magnetic Resonance
PDB: Protein Data Bank
UIPAC: International Union of Pure and Applied Chemistry
