# Supplementary material for: Glycosylator: a Python framework for the rapid modeling of glycans
Source: BMC Bioinformatics. 2019 Oct 22;20:513. doi: 10.1186/s12859-019-3097-6 (PMC6806574; doi:10.1186/s12859-019-3097-6)
Supplement: Supplementary file 1 — Additional file 1: Figure S1. Architecture of Glycosylator, a Python framework for the rapid modeling of glycans. Each class is represented by a hexagon. Full circles connecting classes indicate a class that contains an instance of the previous one as an attribute, e.g. instances of Molecule and MoleculeBuilder are attributes of Glycosylator. Several attributes from Glycosylator can be directly shared with Drawer and Sampler (white squares). Glycosylator can parse a PDB file of a glycoprotein and identify all the sequons (orange rhombus). The glycans (blue squares and green circles) will be extracted and saved as Molecule instances. Glycans at each sequon can then be built, modified or identified. Figure S2. Glycosylator Graphical User Interface. a) The main window is used to import a PDB file of a glycoprotein. Glycosylator will produce a symbolic representation (orange dashed line rectangle). A specific sequon can be selected in the right panel (purple dashed line rectangle). The glycan can be modified by clicking on the symbolic representation. b) The user can select a glycan from the common library or a library that they created. The selected glycan is highlighted with a red square. Example S1. Building a glycan. The structure of an N-Acetyl-D-Glucosamine will be imported as a Molecule instance. All missing atoms will be added according to the CHARMM force field. A second N-Acetyl-D-Glucosamine will then be linked through a 1-4 glycosidic bond. Finally, an Alpha-D-added according to the CHARMM force field. A second N-Acetyl-D-Glucosamine will then be linked through a 1-4 glycosidic bond. Finally, an Alpha-D-Mannosewill be built ab initio and saved to a PDB file. Example S2. Importing, identifying and editing of a glycan. The structure of a mannose 9 will be imported as a Molecule instance. A Glycosylator instance will then identify it against a database of known structures. Finally, the Molecule will be trimmed down to a mannose 6. [file 12859_2019_3097_MOESM1_ESM.docx]

**Glycosylator: A Python framework for the rapid modeling of glycans**

Thomas Lemmin^1,2*^ and Cinque Soto^3,4^*

^1^ System Group, Department of Computer Sciences, ETH Zürich, CH-8093 Zürich, Switzerland.

^2^ Institute of Medical Virology, University of Zurich, CH-8057 Zürich, Switzerland.

^3^Vanderbilt Vaccine Center, Vanderbilt University Medical Center, Nashville, TN 37232, USA.

^4^Department of Pediatrics, Vanderbilt University Medical Center, Nashville, TN 37232, USA.


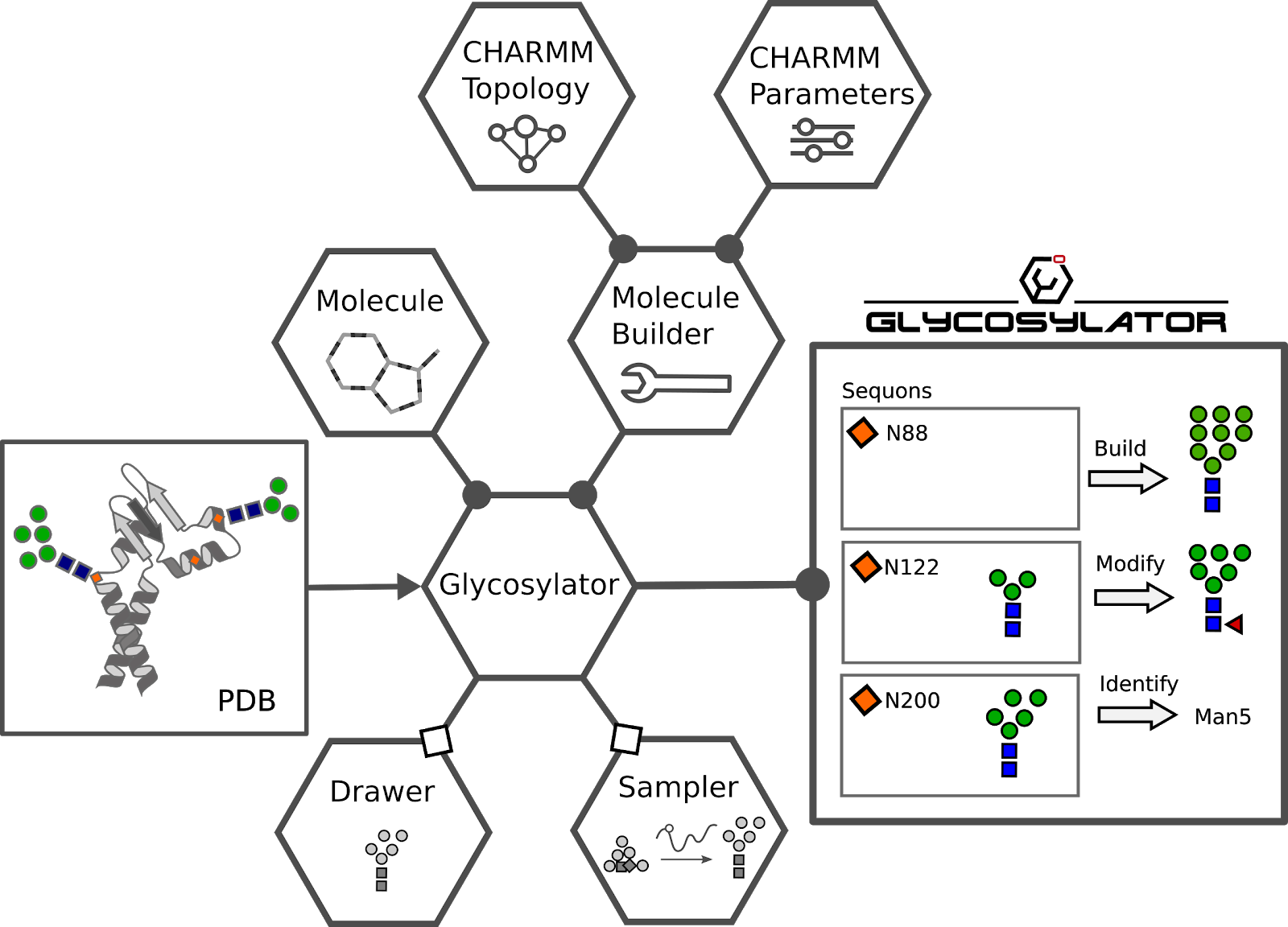


**Figure S1. Architecture of Glycosylator, a Python framework for the rapid modeling of glycans.** Each class is represented by a hexagon. Full circles connecting classes indicate a class that contains an instance of the previous one as an attribute, e.g. instances of Molecule and MoleculeBuilder are attributes of Glycosylator. Several attributes from Glycosylator can be directly shared with Drawer and Sampler (white squares). Glycosylator can parse a PDB file of a glycoprotein and identify all the sequons (orange rhombus). The glycans (blue squares and green circles) will be extracted and saved as Molecule instances. Glycans at each sequon can then be built, modified or identified.


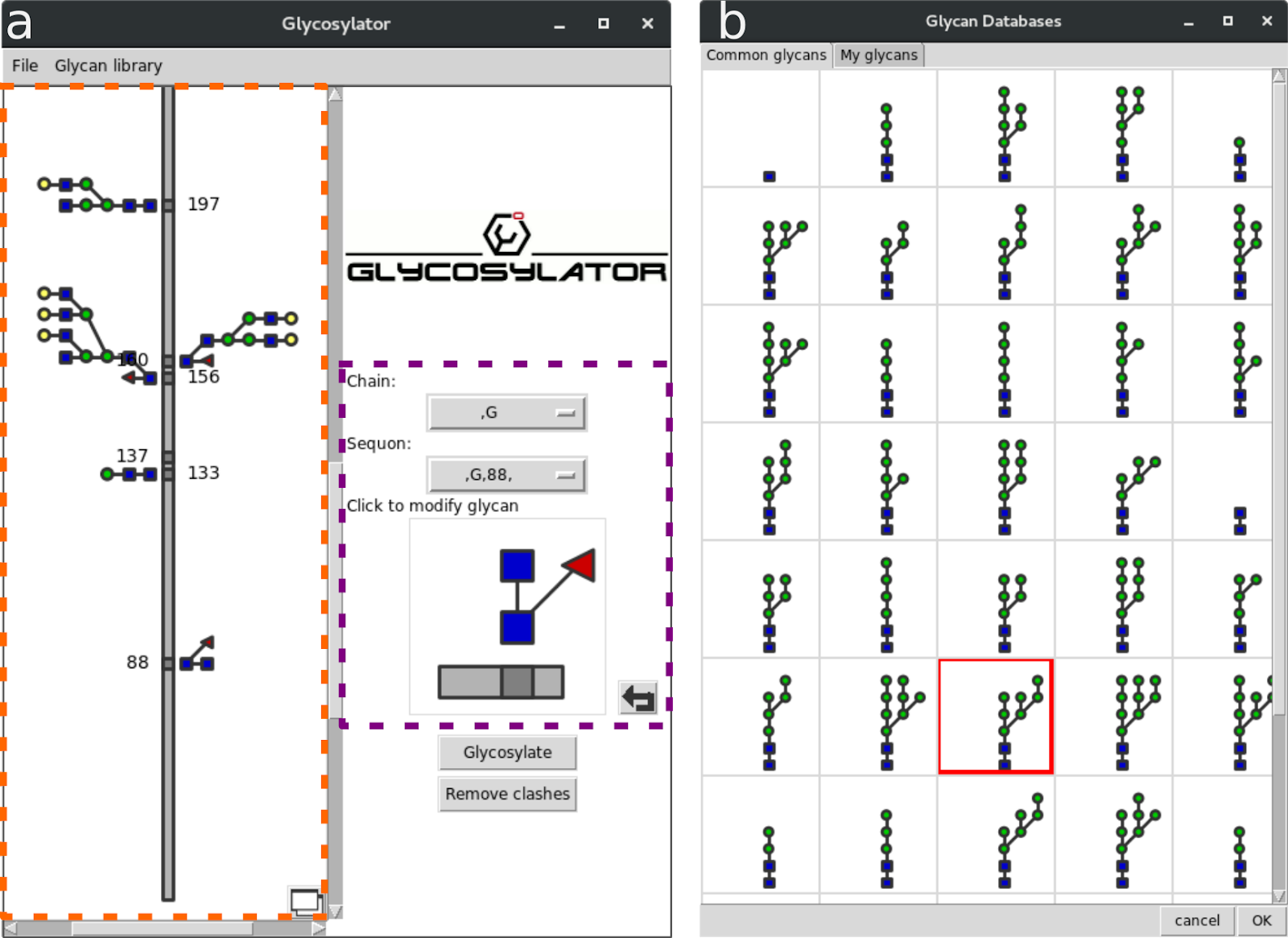


**Figure S2. Glycosylator Graphical User Interface.** a) The main window is used to import a PDB file of a glycoprotein. Glycosylator will produce a symbolic representation (orange dashed line rectangle). A specific sequon can be selected in the right panel (purple dashed line rectangle). The glycan can be modified by clicking on the symbolic representation. b) The user can select a glycan from the common library or a library that they created. The selected glycan is highlighted with a red square.

**Illustrative Examples**

We will present here below two complete examples for building and identifying glycans. An exhaustive list of examples can by found in the demo folder provided with the Glycosylator git repository.

In the first example, the structure of an N-Acetyl-D-Glucosamine will be imported as a Molecule instance. All missing atoms will be added according to the CHARMM force field. A second N-Acetyl-D-Glucosamine will then be linked through a 1-4 glycosidic bond. Finally, an Alpha-D-Mannose will be built *ab initio* and saved to a PDB file.

| #!usr/bin/env python  """ demo_builder.py  The MoleculeBuilder class is used to build ab initio or add missing atoms from a topology file to a molecule. The topology and parameter files are based on CHARMM force field and are parsed with instances of CHARMMTopology and CHARMMParameter  1. The initialization of a MoleculeBuilder requires a topology and parameter file 2. The MoleculeBuilder identifies the missing atoms in a residue (AtomGroup) 3. The internal coordinates (IC) from the topology are used to define the coordinates of missing atoms. The IC should not have a circular dependency. 4. A Molecule can be chemically modified with a patch (CHARMM definition) 5. A Molecule can also be built ab initio using a DUMMY patch """ import glycosylator as gl import prody as pd import os ################################################################### #Create a builder instance # 1. Initialization requires a topology and parameter in CHARMM format myBuilder = gl.MoleculeBuilder(os.path.join(gl.GLYCOSYLATOR_PATH, 'support/toppar_charmm/carbohydrates.rtf'), os.path.join(gl.GLYCOSYLATOR_PATH, 'support/toppar_charmm/carbohydrates.prm')) #################################################################### #Initialize a Molecule instance from the PDB file. (N-Acetyl-D-Glucosamine) myNAG = gl.Molecule('NAG') myNAG.read_molecule_from_PDB(os.path.join(gl.GLYCOSYLATOR_PATH,'support/examples/NAG.pdb')) #################################################################### # 2. Add missing atoms NAG_complete,missing_atoms,bonds = myBuilder.add_missing_atoms(myNAG.atom_group) # 3. Build in the  missing atoms for internal coordinates myBuilder.build_missing_atom_coord(NAG_complete, missing_atoms, myBuilder.Topology.topology['NAG']['IC']) # 4. Save new coordinates and bonds myNAG.set_AtomGroup(NAG_complete, bonds = bonds, update_bonds = False) # 5. Update angles and dihedrals based on bonds myNAG.update_connectivity(update_bonds = False) pd.writePDB('NAG_complete.pdb', NAG_complete) #################################################################### # 6. Connect a second NAG using a 14bb connectivity NAG2, del_atoms, bonds2 = myBuilder.build_from_patch(myNAG.atom_group, 2, 'NAG', myNAG.get_chain(), myNAG.get_segname(), '14bb') NAG_14bb_NAG = myBuilder.delete_atoms(NAG_complete, del_atoms) NAG_14bb_NAG += myBuilder.delete_atoms(NAG2, del_atoms) pd.writePDB('NAG_14bb_NAG.pdb', NAG_14bb_NAG) #####################################################################Build a MAN ab initio #Load patch for building a molecule ab initio (DUMMY) myBuilder.Topology.read_topology(os.path.join(gl.GLYCOSYLATOR_PATH, 'support/topology/DUMMY.top')) Man, del_atoms, bond_man = myBuilder.build_from_DUMMY(1, 'MAN', 'G', '1G', 'DUMMY_MAN') pd.writePDB('MAN_DUMMY.pdb', Man) |
| --- |

In the second example below, the structure of a mannose 9 will be imported as a Molecule instance. A Glycosylator instance will then identify it against a database of known structures. Finally, the Molecule will be trimmed down to a mannose 6.

| #!usr/bin/env python  """ demo_glycosylator.py  The Glycosylator class is used for modelling glycans. The following code will illustrate how to manipulate single glycans 1. The initialization of a glycosylator requires a topology and parameter file 2. Additional topology files can be added 3. A connectivity tree file is used to identify and build glycans. A glycan is composed of a "UNIT" (! are used for comments)     A UNIT is defined by its residue name (3 letter code from PDB), the connecting atom, and the path from the root UNIT (first unit in glycan [connected to sequon])     The path is defined by the patches that have been applied to connect the previous UNIT.      Example for mannose 4:                                       \|-16ab->MAN[Z4]-13ab-> MAN[Z7]   NAG[Z1]-14bb->NAG[Z2]-14bb->BMA[Z3]-\|                                       \|-13ab-> MAN[Z7]  Corresponding connectivity topology: RESI MAN4 !User defined, which has to be unique within a top file UNIT NAG                      !Z1 Root unit in glycan UNIT NAG C1 14bb              !Z2 connected to root with 14bb patch UNIT BMA C1 14bb 14bb !Z3 Unit connected with 14bb to Z2                                    ![path from root: 14bb] UNIT MAN C1 14bb 14bb 16ab      !Z4 Unit connected with 16ab to Z3 UNIT MAN C1 14bb 14bb 16ab 13ab !Z7 Unit connected with 13ab to Z4 UNIT MAN C1 14bb 14bb 13ab      !Z9 Unit connected with 13ab to Z3  4. The connectivity tree is automatically inferred based on bonds and patches. This can be used to identify glycan in the connectivity tree library 5. A template glycan can be modified to match the defined connectivity tree (e.g Mannose 6: MAN6_1;3,2) 6. A topology tree can also be directly defined as a dictionary 7. A glycan can be built ab initio """  import glycosylator as gl import prody as pd import os   #################################################################### # 1. Create a glycosylator myGlycosylator = gl.Glycosylator(os.path.join(gl.GLYCOSYLATOR_PATH,                                  'support/toppar_charmm/carbohydrates.rtf'),                                    os.path.join(gl.GLYCOSYLATOR_PATH,                                   'support/toppar_charmm/carbohydrates.prm')) # 3. Load glycan connectivity tree library myGlycosylator.read_connectivity_topology(os.path.join(gl.GLYCOSYLATOR_PATH,                                           'support/topology/mannose.top'))  #################################################################### # 4. Identify glycan in mannose.top myMan9 = gl.Molecule('mannose9') myMan9.read_molecule_from_PDB(os.path.join(gl.GLYCOSYLATOR_PATH, 'support/examples/man9.pdb'), update_bonds = True) myGlycosylator.assign_patches(myMan9) print "Identified glycan: ", myGlycosylator.identify_glycan(myMan9) #################################################################### # 5. Trim man9 down to man6 (name in database is MAN6_1;3,2) connect_tree = myGlycosylator.build_connectivity_tree(myMan9.rootRes, myMan9.interresidue_connectivity) man6, bonds6  = myGlycosylator.glycosylate('MAN6_1;3,2', template_glycan_tree = connect_tree, template_glycan = myMan9.atom_group) myMan6 = gl.Molecule('mannose6') myMan6.set_AtomGroup(man6, bonds = bonds6, update_bonds = False) |
| --- |
|  |
